# Long-Term Follow-Up of Patients with Catecholaminergic Polymorphic Ventricular Arrhythmia

**DOI:** 10.3390/jcm9040903

**Published:** 2020-03-25

**Authors:** Michael Veith, Ibrahim El-Battrawy, Gretje Roterberg, Laura Raschwitz, Siegfried Lang, Christian Wolpert, Rainer Schimpf, Xiaobo Zhou, Ibrahim Akin, Martin Borggrefe

**Affiliations:** 1 First Department of Medicine, Faculty of Medicine, University Medical Centre Mannheim (UMM), University of Heidelberg, 68167 Mannheim, Germany; gretje.roterberg@web.de (G.R.); laura_raschwitz@yahoo.de (L.R.); Siegfried.Lang@medma.uni-heidelberg.de (S.L.); Christian.wolpert@medma.uni-heidelberg.de (C.W.); rainer.schimpf@medma.uni-heidelberg.de (R.S.); Xiaobo.zhou@medma.uni-heidelberg.de (X.Z.); Ibrahim.Akin@umm.de (I.A.); Martin.borggrefe2006@gmail.com (M.B.); 2 DZHK (German Center for Cardiovascular Research), Partner Site, Heidelberg-Mannheim, 68167 Mannheim, Germany

**Keywords:** CPVT, sudden cardiac death, ventricular tachyarrhythmia, ICD

## Abstract

Background: Catecholaminergic polymorphic ventricular tachycardia (CPVT) is a rare inherited disorder causing life-threatening arrhythmias. Long-term outcome studies of the channelopathy are limited. Objective: The aim of the present study was to summarize our knowledge on CPVT patients, including the clinical profile treatment approach and long-term outcome. Methods: In this single center study, we retrospectively and prospectively collected data from nine CPVT patients and analyzed them. Results: We reviewed nine patients with CPVT in seven families (22% male), with a median follow-up time of 8.6 years. Mean age at diagnosis was 26.4 ± 12 years. Symptoms at admission were syncope (four patients) and aborted cardiac arrest (four patients). Family history of sudden cardiac death was screened in five patients. In genetic analyses, we found five patients with ryanodine type 2 receptor (*RYR2*) mutations. Seven patients were treated with beta-blockers, and if symptoms persisted flecainide was added (four patients). Despite beta-blocker treatment, three patients suffered from seven adverse cardiac events. An implantable cardioverter defibrillator was implanted in seven patients (one primary, six secondary prevention). Over the follow-up period, three patients suffered from ventricular tachycardia (ten times) and five patients from ventricular fibrillation (nine times). No one died during follow-up. Conclusion: Our CPVT cohort showed a high risk of cardiac events. Family screening, optimal medical therapy and individualized treatment are necessary in affected patients in referral centers.

## 1. Introduction

Catecholaminergic polymorphic ventricular tachycardia (CPVT) is an inherited arrhythmogenic disorder characterized by ventricular arrhythmias (VAs) induced by physical or emotional stress without any detectable structural abnormalities of the heart muscle [1,2]. This rare channelopathy manifests especially in childhood and adolescence, with symptoms such as syncope and/or sudden cardiac arrest. The risk of cardiac events is high, and the incidence of sudden cardiac death (SCD) has been estimated to be 13% over an eight-year period [3]. About 60% of CPVT patients are mutation carriers of the gene encoding the cardiac ryanodine type 2 receptor (*RYR2*; CPVT type 1) [4]. *CASQ2* mutations, encoding the calsequestrin 2 gene, are related to CPVT type 2 [5,6]. It has been discussed that these gene mutations trigger ventricular tachyarrhythmias due to calcium leakage in the cardiomyocyte [4]. To minimize cardiac events, beta-blockers can be an effective medical treatment. The VA event rate persists at approximately 25% of CPVT patients despite the beta-blocker therapy [3,7]. Flecainide has been seen as a good option to add for the reduction of VAs [8]. Besides medical treatment, an implantable cardioverter defibrillator (ICD) is often used in patients with refractory ventricular tachyarrhythmias to prevent SCD [9,10]. Furthermore, left cardiac sympathetic denervation (LCSD) could be an effective anti-fibrillatory intervention for patients with recurrent ventricular tachyarrhythmias, in addition to medical therapy and/or ICD implantation [11]. In this study, we report the clinical characteristics, genetic profile, treatment and long-term follow-up of a CPVT cohort, including family members.

## 2. Methods

In this retrospective and prospective study, we describe 7 CPVT families. The data were obtained from existing medical records. Medical records included symptoms, family screening, electrocardiograms (ECGs), medical treatment, ICD surveillance, electrophysiological studies, exercise stress testing, echocardiography, cardiac magnetic resonance imaging (MRI), cardiac computed tomography (CT) and genetic analyses. To evaluate the event rate, patients were contacted to reassess their symptoms and treatment. If patients were not reached, their physicians and/or their relatives were contacted. The diagnosis of CPVT was defined according to the ESC (European Society of cardiology) criteria in 2015. The following two recommendations are described there: (1) CPVT is diagnosed in the presence of a structurally normal heart, normal ECG and exercise—or emotion-induced bidirectional or polymorphic ventricular tachycardia. (2) CPVT is diagnosed in patients who are carriers of a pathogenetic mutation in the *RYR2* and *CASQ2* genes [12]. To exclude structural heart disease echocardiography, cardiac computer tomography (CT) and cardiac magnetic resonance imaging (MRI) were performed. Family screening was performed. We contacted the patients, asked them about their medical family history and if any heart diseases or symptoms occurred in the pedigree. The Declaration of Helsinki was complied with, and the study protocol was confirmed by the Ethics Committee of the Medical Faculty Mannheim (ethical approval code: 2018-877R-MA).

### 2.1. Definitions

Syncope was defined as loss of consciousness induced by sport or emotion. Cardiac events were syncope, aborted cardiac arrest (ACA), non-aborted sudden cardiac death (SCD) and appropriate ICD shock. SCD means death by cardiac arrest in a healthy person. Ventricular arrhythmias (VA) could be non-sustained ventricular tachycardia (VT), bidirectional sustained VT or ventricular fibrillation (VF). Ventricular extrasystole (VES) was ventricular extrasystole in the electrocardiogram. If the ICD detected a VT that terminated spontaneously and/or the patient had no symptoms, it was defined as a VA and not a cardiac event. Sinus bradycardia is a heart rate lower than 60 bpm. An exercise stress test was performed in every patient to evaluate the diagnosis of CPVT. Asymptomatic patients are those without any cardiac events. Symptomatic patients are patients with cardiac events, including syncope.

### 2.2. Genetic Screening

For the genetic screening of CPVT1 (associated with *RYR2*) and CPVT2 (associated with *CASQ2*), DNA was analyzed. The blood samples were sent to a human genetics laboratory. CPVT was diagnosed by at least two independent, experienced cardiologists after diagnostic findings.

### 2.3. Statistics

Tables present categorical variables as frequency (percentage). For continuous variables, mean ± SD or median (interquartile range) were used as appropriate, and categorical variables were presented as numbers (percentage). A Kaplan–Meier curve was created to show the risk of cardiac events with age. Statistical analysis was completed with SPSS statistics 23.0 (Armonk, NY, USA; IBM Corp) in all analyses.

## 3. Results

### 3.1. Demographics, Clinical Profile and Follow-Up Data

A total of nine patients were diagnosed with CPVT. It occurred in seven independent families. Baseline characteristics are illustrated in Table 1. Of the patients, 22% were male and 78% female. Mean age at onset of symptoms was 20.4 ± 10.3 years, and the mean age at diagnosis was 26.4 ± 12 years. Only one patient was diagnosed with CPVT 10 months after the onset of symptoms; all others had more than a one-year delay in diagnosis. None of them were misdiagnosed with epilepsy. The reason for admission was syncope in 4 (44%) patients and ACA in 4 (44%) patients. One patient was asymptomatic at clinical presentation, and three (33%) had atrial fibrillation. Family screening revealed five SCD cases and three other patients with CPVT. An ECG at rest at admission was available in seven patients, and the heart rate was 52.9 ± 6 bpm. An exercise stress test was performed in all patients to confirm the diagnosis. In one patient, the induction of VES as well as VT was shown, and in the other seven patients only VES were documented. Only in one patient were no VES and/or ventricular tachyarrhythmias induced. Beta-blockers were started in seven (78%) patients, and if there were still symptoms after the maximum tolerated dose, flecainide was added (44%) (Table 2). LCSD was not undertaken in any patient. Seven patients (78%) underwent ICD implantation. An exercise stress test was performed in three patients prior to ICD implantation. One person had an event recorder.

### 3.2. Symptoms Per Family

In the seven CPVT families, the following symptoms occurred: Syncope (four families), ACA (four families), atrial arrhythmias (three families), non-aborted SCD (four families), and affected genes were documented in three families.

### 3.3. Genetic Screening

The details of genetic screening are shown in Table 2. The following three different mutations were found in the *RYR2* gene consistent with CPVT type 1: patient 2 and patient 3 with mutation (D2216G), patient 4 and patient 5 with mutation (L2432F), and patient 6 with mutation (M4002V). No mutations were identified in three patients.

### 3.4. Detailed Description of CPVT Families

#### 3.4.1. Family 1

The clinical presentation of patient 1 was VF while she was doing sport at the gym. She was resuscitated, and a single-chamber ICD was implanted for secondary prevention. Echocardiography, cardiac CT and MRI showed no structural heart disease. After diagnosis, we initiated the patient on metoprolol. Because of fatigue, and blue lips and fingers, the patient stopped the medical therapy after 8 months. The consequence of this was more palpitations and supraventricular tachycardias (SVT) being observed. In the time without any medication, she received two inappropriate shocks due to SVTs, while she was doing sport. Verapamil was taken for 21 months to suppress the SVTs. There were no cases of SCD in her family, and her daughter had no symptoms yet.

#### 3.4.2. Family 2

**Figure 1:**
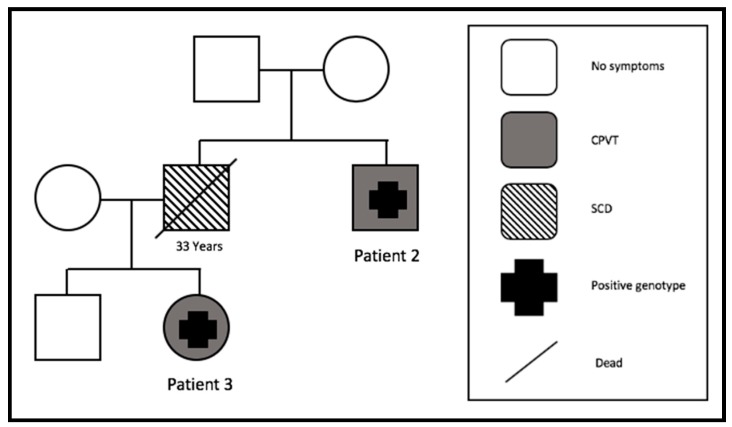
Pedigree of family 2. One SCD at the age of 33 years (SCD = sudden cardiac death). CPVT = catecholaminergic polymorphic ventricular tachycardia.

Our oldest patient (patient 2), who was 42 years old at his first symptoms, was admitted with recurrent syncope after emotional stress. Since then, he had three more syncopes. His brother died suddenly at the age of 33 (Figure 1); therefore, a transvenous ICD implantation for primary prevention was recommended. Echocardiography and cardiac MRI excluded structural heart problems. A subcutaneous ICD was implanted four months later. Five years after ICD implantation, he was shocked two times inappropriately because of an electrode isolation defect. No cardiac events appeared after ICD implantation.

His niece (patient 3) had the same *RYR2* mutation. While she was swimming, VF was documented and she had an ACA at admission. Echocardiography and cardiac MRI were negative for structural heart problems. A submuscular ICD was implanted, and nine months later she received an appropriate ICD shock and terminated a VF episode, while she was doing sport. One more VF episode terminated spontaneously on this day. After this cardiac event, bisoprolol therapy was started for two months and then switched to metoprolol. During follow-up, there was one electrode defect four years after implantation. No more cardiac events occurred since starting the patient on beta-blockers.

#### 3.4.3. Family 3

**Figure 2:**
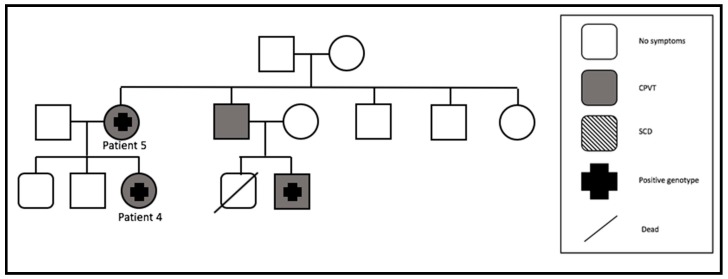
Pedigree of family 3 (SCD = sudden cardiac death).

One patient was asymptomatic during the whole follow-up, as well as at clinical presentation (patient 5). She stopped beta-blocker therapy right after initiation, because of side effects and no cardiac symptoms. She was a carrier of the *RYR2* mutation (Figure 2). Her daughter (patient 4), also with this mutation, presented with syncope. Echocardiography and cardiac MRI showed no structural heart problems in either of the patients. Bisoprolol therapy was initiated in patient 4. Despite this therapy, two syncopes occurred, a subsequent ICD was implanted, and flecainide was started. No more events were documented. Two more family members were detected with CPVT, but the brother of patient 5 refused genetic screening, although he was symptomatic with one syncope. The brother of patient 5 had a son, who also had a mutation in *RYR2* and a syncope. No further information and follow-up are available for them, because it was impossible to contact them again. We lost the whole family (patient 4 and 5) to our follow-up.

#### 3.4.4. Family 4

**Figure 3:**
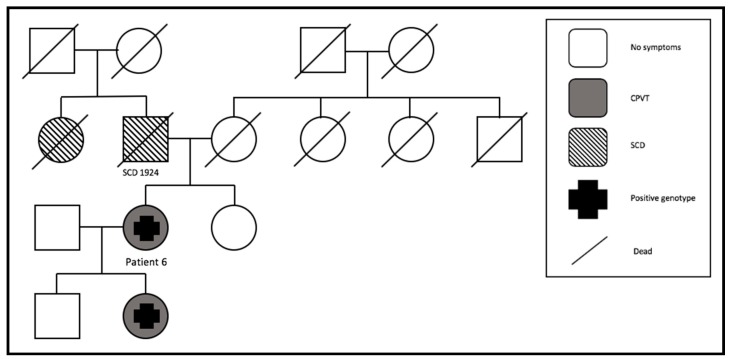
Pedigree of family 4. Two SCD cases (SCD = sudden cardiac death).

Patient 6 had her first syncope at the age of 11. After that, a syncope occurred every year. A few years later she suffered an ACA, and an ICD was implanted. At this moment, we also started a beta-blocker (metoprolol). Since then, no more cardiac events occurred. We had to replace the aggregate because of battery depletion two times during the whole follow-up, but no complications appeared. Eleven years after the initiation of metoprolol, we added flecainide because of recurrent palpitations. During pregnancy, the beta-blocker dosage was reduced and flecainide was stopped. The ICD promptly shocked her two times due to sustained VT. The pedigree showed that she was affected by the *RYR2* mutation, as well as her daughter (Figure 3). Her daughter was asymptomatic. In the family history, two cases of SCD were documented.

#### 3.4.5. Family 5

The clinical presentation of patient 7 was ACA, because of polymorphic VT and VF during sport. For secondary prevention, we implanted an ICD (transvenous). Structural heart disease was excluded. The patient suffered frequently from inappropriate ICD shocks (*n* = 4), which appeared because of atrial fibrillation. Therefore, propafenone was started for 10 years. The genetic screening was negative for *RYR2*, and no relatives were known to have experienced SCD. We stopped propafenone, started metoprolol monotherapy for one month and added flecainide to the beta-blocker. In the exercise stress test, she suffered a non-sustained polymorphic VT. No cardiac events occurred during the follow-up.

#### 3.4.6. Family 6

**Figure 4:**
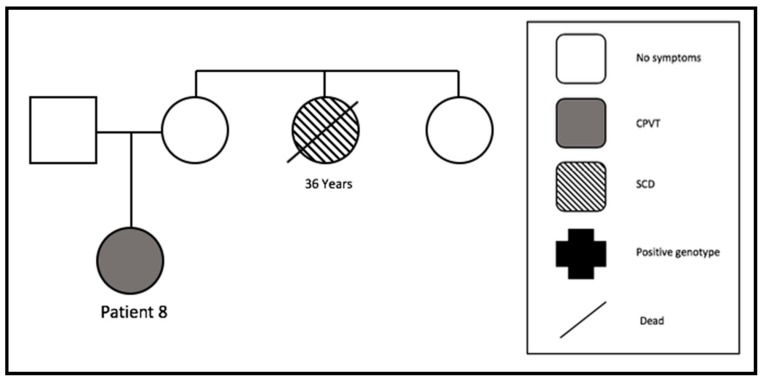
Pedigree of family 6. One SCD at the age of 36 years (SCD = sudden cardiac death).

While patient 8 received a rhinoplasty, a bidirectional VT occurred while she was under anesthesia. Before this event, she received six stress- and sport-associated syncopes. The echocardiography and cardiac MRI were negative for structural heart disease. After the initiation of metoprolol, three more syncopes occurred; however, the compliance to beta-blocker intake was uncertain. We implanted an event recorder in her. She stopped taking metoprolol because of fatigue and dizziness. After that, she rejected other medication. No arrhythmic events were documented, so she decided to explant the recorder. Her aunt died suddenly at the age of 36. No further cases of SCD were detected in this family (Figure 4).

#### 3.4.7. Family 7

**Figure 5:**
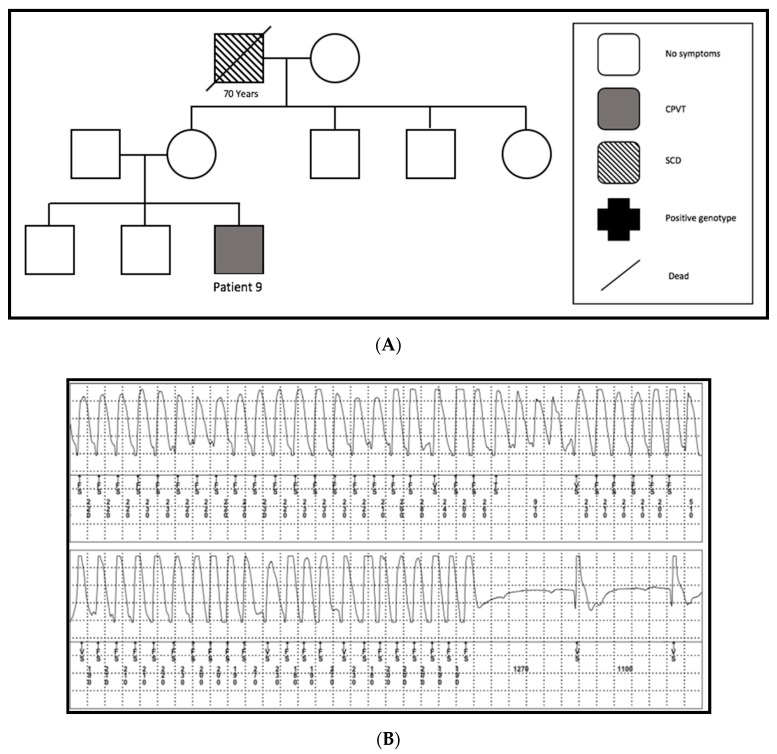
(**A**) Pedigree of family 7. One SCD at the age of 70 years (SCD = sudden cardiac death). (**B**) A representative chart of ventricular tachycardia of patient number 9 documented in his implanted event recorder during football.

A 27-year-old patient (patient 9) was admitted to the hospital because of two syncopes while he was playing football. Echocardiography as well as cardiac MRI excluded structural heart problems. Genetic screening could not detect any mutation. We detected one SCD in his family (Figure 5A). The diagnosis was made two years later, after he had three more syncopes due to VF. These were documented in an event recorder during sport (Figure 5B). A subcutaneous ICD was implanted and the patient was started on propanolol. Because of noncompliance, the patient stopped the drug one month later. Since the ICD implantation, he had no cardiac events and/or ICD complications.

### 3.5. Long-Term Follow-Up

All patients also had periods without medical treatment, because of noncompliance or due to being between the time of admission and diagnosis. The mean follow-up without medication was 5.1 years, during which time four patients had eight syncopes and six VFs.

Despite beta-blocker treatment in seven patients (4 years mean follow-up time), three patients suffered five syncopes and two VTs. Flecainide follow-up was available in three patients over a 5.7-year mean period. It was added because of recurrent palpitations (patient 6) and a high rate of VES in ergometry (patients 4 and 7). In this time, no cardiac events occurred. 

The ICD and event recorder follow-up (8.8 years mean) for four patients showed four syncopes, two VFs and eight VTs.

**Figure 6:**
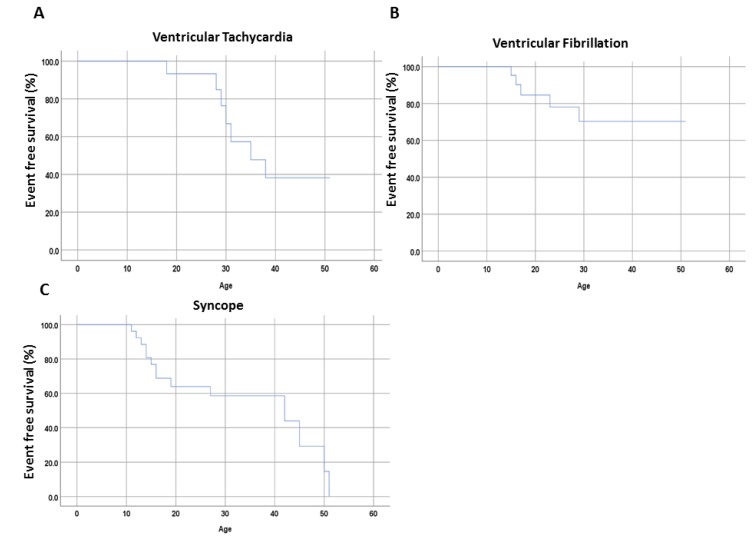
Kaplan-Meier curvesillustrate the proportion of cardiac event free timein the families with CPVTof the present study. (**A**) Ventricular Tachycardia; (**B**) Ventricular Fibrillation; (**C**) Snycope.

Median follow-up time over the whole cohort was 8.6 years (IQR 6.3–17.4). Figure 6 presents the incidence of VT, VF and SCD. Over the follow-up period, three patients suffered from VT (*n* = 10), and five patients from VF (*n* = 9). No one died during the entire follow-up.

## 4. Discussion

We have described the clinical profile and short- and long-term risk of cardiac events in seven CPVT families and found the following: (i) the risk of cardiac events in CPVT families is high; (ii) ICD implantation is recommended in high-risk patients; (iii) family screening is essential to detect all CPVT patients; (iv) the outcome is improved by taking appropriate risk assessment, individualized treatment options (beta-blocker/flecainide and/or ICD) and if the patient is seen at a reference center.

Our data present one of the longest long-term follow-up studies in CPVT patients.

CPVT is an inherited channelopathy associated with a substantial risk of SCD. The resting ECG is typically normal, although 18% of the mutation-positive patients have sinus bradycardia [13]. Our data have also shown the same phenomenon in the majority of patients. To support the diagnosis of CPVT, an exercise stress test has to be performed, but it is often unreliable because many patients are exhausted before the maximum workload is achieved and in many cases, it can simply trigger VES [3,12,13]. The performed exercise stress test in our cohort evidenced in one patient neither VT nor VES, and in total just one case of VT appeared. Consequentiallythe diagnosis often occurred more than a yearafter onset of symptoms [3,14].

The presence of an *RYR2* mutation has been described in up to 60% of published CPVT families [4]. Here, we describe an *RYR2* mutation rate of up to 43% of our families and 56% of our cohort. None of our patients presented mutations in *CASQ2*. CALM1-3, SCN5A and TRDN have also been implicated in CPVT [15,16,17]. However, recently published data have described a new genetic association of CPVT in the trans-2, 3-enoyl-CoA reductase-like (TECRL) gene [18]. TECRL homozygous c.331 + 1G >, a splice site mutation in iPSCs (induced pluripotent stem cells), revealed a definite correlation between TECRL and Ca^2+^ transport in cardiomyocytes [18].

Since understanding the function of the adrenergic mechanism of cardiac events in CPVT, beta-blockers are the first-line therapy [1,3,19]. Despite using of beta-blockers complications like breakthrough VA, noncompliance, underdosing and/or side effects are still considerably [7]. During follow-up, three patients treated with beta-blockers had seven cardiac events, including two VTs due to a lower dosing because of pregnancy. For those in our cohort without any medication, 19 cardiac events appeared in four patients. Thus, we can record an obvious improvement of cardiac events after beta-blocker treatment. Unfortunately, the compliance of our patients was not perfect. When they were symptomatic and had a recent event, all took their medication. However, one third stopped taking beta-blockers after a while, because they seemed to be asymptomatic and the medication was making them dizzy and tired.

Consensus guidelines recommend ICD in patients with syncope or cardiac arrest, despite optimal drug therapies [12]. ICDs have been shown to rescue patients with CPVT from cardiac arrest. About 47% of patients received an ICD for primary prevention, and during follow-up 40% had an appropriate shock as published by a study in 2018 [20]. The survival of undiagnosed CPVT patients with ICDs who presented with cardiac arrest was not improved [21]. Our cohort showed 14% of patients with ICD for primary prevention. In 29% of our patients 4 appropriate shocks due to ventricular tachyarrhythmias were observed.

## 5. Conclusions

Our data present a cohort of nine CPVT patients among seven families. Most patients with CPVT presented with symptoms such as syncope or ACA. Family screening is necessary in affected patients. Optimal medical therapy is paramount in preventing ventricular tachyarrhythmias. Thus, long-term outcomes can be improved by individualized treatment.

## Figures and Tables

**Table 1:** Baseline characteristics of catecholaminergic polymorphic ventricular tachycardia (CPVT) patients.

Variables	*N* = 9
Demographics	
Age at first symptoms, mean ± SD	20.4 ± 10.3
Age at diagnosis, mean ± SD	26.4 ± 12
Male, *n* (%)	2 (22.2)
Symptoms at admission, *n* (%)	
Syncope	4 (44.4)
Aborted cardiac arrest	4 (44.4)
Atrial arrhythmias	3 (33.3)
Family history of SCD	5 (55.6)
Epilepsy	0 (0)
ECG at admission	
Sinus rhythm	9 (100)
Exercise stress test, *n* (%)	
Induction of VT or VF	1 (11.1)
Induction of VES	8 (88.9)
Genetic screening, *n* (%)	
RYR2	5 (55.6)
CASQ2	0 (0)
Not identified	3 (33.3)
Not screened	1 (11.1)
Treatment, *n* (%)	
Beta-blocker	7 (77.8)
Flecainide and Beta-blocker	4 (44.4)
LCSD	0 (0)
ICD Implantation, *n* (%)	
Yes	7 (77.8)
No	1 (11.1)
Event recorder	1 (11.1)

SCD = sudden cardiac death; VT = ventricular tachycardia; VF = ventricular fibrillation; VES = ventricular extrasystole; RYR2 = ryanodine type 2 receptor; CASQ2 = calsequestrin 2; LCSD = left cardiac sympathetic denervation; ICD = implantable cardioverter defibrillator.

**Table 2:** Details of the patients.

Patient Number	1	2	3	4	5	6	7	8	9
Sex	female	male	female	female	female	female	female	female	male
Age at diagnosis (years)	23	55	16	13	20	31	33	18	29
Family number	1	2	2	3	3	4	5	6	7
Gene mutation (mutation sequence)	not screened	RYR2 (D2216G)	RYR2 (D2216G)	RYR2 (L2432F)	RYR2 (L2432F)	RYR2 (M4002V)	0	0	0
Initial medical treatment	Metoprolol 50 mg/day	no medical treatment	Bisoprolol 2.5 mg/day	Bisoprolol 2.5 mg/day	no medical treatment	Metoprolol 200 mg/day	Propafenone 450 mg/day	Metoprolol 150 mg/day	Propanolol (dosage not available)
Changed medical treatment	refused	no medical treatment	Metoprolol 25 mg/day	Bisoprolol 5 mg/day	no medical treatment	Metoprolol 200 mg/day + Flecainide 200 mg/day	Metoprolol 100 mg/day	Metoprolol 150 mg/day	refused
Current medical treatment	refused	no medical treatment	Metoprolol 100 mg/day	Bisoprolol 5 mg/day + Flecainide 200 mg/day	no medical treatment	Metoprolol 200 mg/day + Flecainide 200 mg/day	Metoprolol 100 mg/day + Flecainide 100 mg/day	refused	refused
